# Altered Structural Covariance of Insula, Cerebellum and Prefrontal Cortex Is Associated with Somatic Symptom Levels in Irritable Bowel Syndrome (IBS)

**DOI:** 10.3390/brainsci11121580

**Published:** 2021-11-29

**Authors:** Cecilia Grinsvall, Lukas Van Oudenhove, Patrick Dupont, Hyo Jin Ryu, Maria Ljungberg, Jennifer S. Labus, Hans Törnblom, Emeran A. Mayer, Magnus Simrén

**Affiliations:** 1 Department of Molecular and Clinical Medicine, Institute of Medicine, Sahlgrenska Academy, University of Gothenburg, 40530 Gothenburg, Sweden; hans.tornblom@gu.se (H.T.); magnus.simren@medicine.gu.se (M.S.); 2 Department of Anaesthesiology and Intensive Care Medicine, Institute of Clinical Sciences, Sahlgrenska Academy, University of Gothenburg, 40530 Gothenburg, Sweden; 3 Department of Anaesthesiology, Intensive Care Medicine/Pain Centre, Sahlgrenska University Hospital, Region Västra Götaland, 41685 Gothenburg, Sweden; 4 Translational Research Center for Gastrointestinal Disorders (TARGID), Laboratory for Brain-Gut Axis Studies (LaBGAS), KU Leuven, 3000 Leuven, Belgium; lukas.vanoudenhove@kuleuven.be; 5 Leuven Brain Institute, KU Leuven, 3000 Leuven, Belgium; patrick.dupont@kuleuven.be; 6 Cognitive & Affective Neuroscience Lab, Department of Psychological & Brain Sciences, Dartmouth College, Hanover, NH 03755, USA; 7 Laboratory for Cognitive Neurology, Department of Neurosciences, KU Leuven, 3000 Leuven, Belgium; 8 School of Osteopathic Medicine in Arizona (ATSU-SOMA), A.T. Still University, Mesa, AZ 85206, USA; hyojinryu@atsu.edu; 9 Department of Radiation Physics, Institute of Clinical Sciences, Sahlgrenska Academy, University of Gothenburg, 41345 Gothenburg, Sweden; maria.ljungberg@vgregion.se; 10 Department of Medical Physics and Biomedical Engineering, Diagnostic Imaging, MR Centre, Sahlgrenska University Hospital, 41345 Gothenburg, Sweden; 11 G. Oppenheimer Center for Neurobiology of Stress & Resilience, Division of Digestive Diseases, David Geffen School, University of California, Los Angeles, CA 90032, USA; jlabus@g.ucla.edu (J.S.L.); emayer@g.ucla.edu (E.A.M.); 12 Center for Functional Gastrointestinal and Motility Disorders, University of North Carolina, Chapel Hill, NC 27599, USA

**Keywords:** irritable bowel syndrome, somatization, central sensitization, brain morphometry, structural covariance, brain imaging

## Abstract

Somatization, defined as the presence of multiple somatic symptoms, frequently occurs in irritable bowel syndrome (IBS) and may constitute the clinical manifestation of a neurobiological sensitization process. Brain imaging data was acquired with T1 weighted 3 tesla MRI, and gray matter morphometry were analyzed using FreeSurfer. We investigated differences in networks of structural covariance, based on graph analysis, between regional gray matter volumes in IBS-related brain regions between IBS patients with high and low somatization levels, and compared them to healthy controls (HCs). When comparing IBS low somatization (N = 31), IBS high somatization (N = 35), and HCs (N = 31), we found: (1) higher centrality and neighbourhood connectivity of prefrontal cortex subregions in IBS high somatization compared to healthy controls; (2) higher centrality of left cerebellum in IBS low somatization compared to both IBS high somatization and healthy controls; (3) higher centrality of the anterior insula in healthy controls compared to both IBS groups, and in IBS low compared to IBS high somatization. The altered structural covariance of prefrontal cortex and anterior insula in IBS high somatization implicates that prefrontal processes may be more important than insular in the neurobiological sensitization process associated with IBS high somatization.

## 1. Introduction

Irritable bowel syndrome (IBS) is a prevalent disorder of brain-gut-interactions [1] characterized by chronic, recurrent abdominal pain and altered bowel habits [2], often accompanied by comorbid psychiatric and other chronic pain disorders [3]. Somatization has been used to refer to the presence of multiple ‘medically unexplained’ symptoms (also known as ‘functional’ symptoms [4]), often assumed to be associated with psychological distress [5]. High levels of somatization, as measured by the Patient Health Questionnaire (PHQ)-12, are more prevalent in IBS patients compared to patients with gastrointestinal complaints not fulfilling IBS criteria [6]. Somatization, defined and measured as the presence and severity of multiple somatic symptoms in IBS, is associated with lower quality of life [7], visceral hypersensitivity [8], postprandial [9], and general gastrointestinal [10] symptoms.

Neuroplasticity refers to structural changes that occur in the adult brain in response to the external environment or internal milieu [11]. There is mounting evidence for structural plasticity and reorganisation in human chronic pain in general [12,13] as well as in IBS specifically [14,15,16,17,18]. Some of the neuroanatomical findings in IBS seem to be specific to the disorder, whereas other differences appear in several chronic pain disorders, and are hence not specific to IBS [19]. IBS is, like other functional somatic syndromes, heterogeneous, and the presence of co-morbid functional disorders and/or somatization may reflect a hypothesized but incompletely understood central sensitization mechanism in this subgroup [20,21].

Analyzing the brain networks with graph analysis is thought to capture neurobiologically important aspects of the organization of brain networks [22]. Abnormal connectivity has been found in patients with neurological and psychiatric disorders compared to healthy controls (HCs) by comparing structural or functional brain network properties, using complex network analysis [23]. More specifically, various neurological and psychiatric conditions have been associated with abnormal structural co-variance networks, including Alzheimer’s disease, schizophrenia, epilepsy and autism [24]. Among the disorders of gut-brain interaction, altered structural covariance of the striatum has been found in functional dyspepsia patients compared to HCs [25]. Only two studies to date have been published using graph analysis on structural brain MRI data in IBS [17,26]: one using binary graphs of regional gray matter volume comparing IBS to HCs [17], and one using weighted graphs of diffusion weighted imaging to study connectivity comparing patients with ulcerative colitis, IBS and HCs [26].

The overall aim of this study was to identify a structural brain network associated with somatization in IBS. We used graph analysis to map the differences in structural gray matter covariance patterns between IBS patients with high and low somatization levels, as well as compare these groups with HCs.

We hypothesized differences in networks of structural covariance between all three groups, greater between IBS high somatization and HCs than between IBS low somatization and HCs, indicative of greater central plastic changes in IBS high compared to low somatization. Based on the previous study by Labus et al., [17], we expected differences between IBS patients and HCs in local but not global graph measures. Based on a study comparing chronic pelvic pain patients with widespread vs. localized symptoms (corresponding to high versus low somatization in our study) [27], we hypothesized that differences in networks of structural covariance between IBS patients with high and low somatization would primarily be found in sensorimotor regions, cingulate cortex, inferior parietal cortex, frontal regions and insula. These regions have shown alterations in structural or functional imaging studies comparing IBS patients with healthy controls without consideration of somatization level [17,19,28].

## 2. Materials and Methods

### 2.1. Subjects

Seventy-seven IBS patients were recruited at the gastroenterology outpatient clinic specializing in functional GI disorders at Sahlgrenska University Hospital in Gothenburg, Sweden, between 2011 and 2014. The IBS patients (18–65 years) came through self-referral or were referred by other physicians, mostly primary care doctors. The IBS diagnosis was based on clinical presentation, fulfilment of the Rome III criteria for IBS [29] and additional investigations if considered necessary by the gastroenterologist (HT or MS). Exclusion criteria included abnormal results on standard screening laboratory tests, severe psychiatric, systemic or other GI diseases, history of drug or alcohol abuse, and the inability to reliably respond to questionnaires in Swedish. The use of probiotics was not allowed during the study period, as it was part of a larger study mapping different pathophysiological mechanisms in IBS, including microbiota. HCs were recruited by local advertisement. The same exclusion criteria as for IBS patients applied for HCs, as well as the presence of IBS according to the Rome III criteria, or reporting more than one mild GI symptom on a GI-symptom screening questionnaire. The study protocol was approved by the Regional Ethical Review Board in Gothenburg (application number 731-09 approved 25 January 2010, with an amendment T240-11 approved 3 March 2011) prior to the start of patient inclusion, and all participants gave their informed consent to participate after verbal and written information. The work in this article have been carried out in accordance with The Code of Ethics of the World Medical Association (Declaration of Helsinki). This study population has also been used to study resting state fMRI connectivity [30], and associations between regional gray matter metrics with visceral sensitivity measures [31].

### 2.2. Questionnaires

PHQ-15 is a validated questionnaire to assess the severity of the 15 most common somatic symptoms; nausea, abdominal pain, altered bowel habit, back pain, limb pain, headaches, chest pain, dizziness, fainting spells, palpitations, breathlessness, menstrual cramps, dyspareunia, insomnia, and lethargy [32]. The score ranges between 0 and 30 and can be used as a continuous measure. Alternatively, cut-off scores of 5, 10, and 15 can be used to define low, medium, and high levels of somatic symptom severity (i.e., somatization) [32]. One of the questions is on menstrual pain and, hence, applies to women only. We removed this question in order not to induce a systematic difference in somatization levels between men and women, and denoted this reduced questionnaire PHQ-14.

The Hospital Anxiety and Depression scale (HADS) is a self-report questionnaire consisting of 14 questions to assess emotional and cognitive aspects of depression and anxiety [33]. We used the total score to denote level of psychological distress [34]. This total HADS score was used solely to compare levels of psychological distress between the three groups.

The IBS severity scoring system (IBS-SSS) is a validated questionnaire to assess IBS symptom severity, consisting of 5 questions including pain severity, frequency of pain, severity of abdominal distension, bowel habit dissatisfaction and how much IBS interferes with life in general [35]. In this study, we used the IBS-SSS solely to characterize the patients and compare between IBS patients with high and low levels of somatization.

### 2.3. Brain Imaging Acquisition

Brain images were acquired on a 3 Tesla Philips Achieva MR scanner using the standard 8 channel head coil. A T1-weighted 3D TFE gradient echo high resolution structural scan was acquired using a magnetization-prepared rapid acquisition gradient echo (MP-RAGE) sequence, with TR = 7.0 ms, TE = 3.2 ms, flip angle = 9°, inversion recovery delay of 900 ms, shot interval of 2200 ms and a bandwidth of 241 Hx/pixel. The acquired and reconstructed voxel size was 1 × 1 × 1 mm^3^, FOV 256 × 220 × 176 mm^3^ and the slice orientation was transverse. No SENSE acceleration was used, however the SENSE-reference scan was used for homogeneity correction of the signal from the individual coil elements.

### 2.4. Structural MRI Analysis

Data processing workflows were designed and implemented at the Laboratory of Neuroimaging (LONI) Pipeline (http://pipeline.loni.usc.edu) as described in Labus et al., 2014 [17]. After quality control, FreeSurfer [36,37] was used for regional parcellation according to the Destrieux cortical atlas and FreeSurfer subcortical stream [38,39,40]. Quality control was based on various indicators of scan quality, including the absence of severe noise and artefacts, and correct segmentation of gray and white matter. Any scans demonstrating pathologies or abnormalities that were deemed sufficiently harmful to structural measures were omitted from further analyses. In total, the brains were parcellated and segmented into two hemispheres with 74 cortical regions each, as well as 15 subcortical regions and two hemispheres of the cerebellum, resulting in a total of 165 brain regions. For this study, the only morphometric measurement used was gray matter volume.

### 2.5. Regions of Interest (ROIs)

Regions that have been consistently shown to be involved in IBS in functional and structural brain imaging were selected as regions of interest (ROIs) [19,28]. As a reference, we used the meta-analysis of fMRI studies of rectal distension by Tillisch et al., (Tables 3 and 4 in [28]) as well as a more recent review of neuroimaging studies in IBS (Table 1 in [19]). This resulted in a total of 18 ROIs: anterior cingulate cortex, midcingulate cortex, amygdala, hippocampus, hypothalamus, anterior insula, middle insula, posterior insula, prefrontal cortex, precentral gyrus, postcentral gyrus, supplementary motor area, thalamus, putamen, cerebellum, midbrain, superior temporal gyrus and inferior parietal lobule. Ninety of the 165 parcellated regions (see Supplementary Materials for details) were determined to correspond to, or be part of, these ROIs.

### 2.6. Data Analysis

#### 2.6.1. Node Definition

The 90 resulting (sub)regions (Supplementary Table S1), which do not overlap, were used as nodes of the network, normalized based on the total gray matter volume (TGMV) prior to analysis. The TGMV was chosen as it contains some information about brain size, sex (female generally smaller) and age (older generally smaller). Therefore, we did not additionally control for sex and age to avoid overcorrecting as well as potential multicollinearity.

#### 2.6.2. Groups

We compared three groups: HCs, IBS patients with low somatization (IBS low somatization) and IBS patients with high somatization (IBS high somatization), with the latter groups defined by a mean split of the PHQ-14 score. The mean PHQ-14 in the full IBS cohort was 12.9, corresponding to a moderate level of somatization according to the PHQ-15 cut-off levels [32]. The mean was used instead of the median, as seven subjects had the median value of PHQ-14 (median = 13), and would have been randomly assigned to one of both groups when using a median split.

#### 2.6.3. Networks of Structural Covariance

In each region (node), we constructed a vector in which each element represents the (TGMV corrected) gray matter volume of a subject. The structural covariance between two nodes was defined as Fisher r-to-Z transformed Pearson correlation coefficient between the two corresponding vectors. Structural covariance was obtained for all pairs of nodes for the three different groups (IBS high somatization, IBS low somatization and HCs).

To assess whether there were significant differences in structural covariance between any pair of regions between the groups, we performed a non-parametric test based on permutation labelling (5000 randomizations) of group membership.

#### 2.6.4. Graph Analysis

To create the weights of the graph (i.e., the connection strength), the Z-matrix of the structural covariance network was used and first transformed into a new matrix which only contained the positive Z values and setting all negative Z values to 0. This new matrix was transformed to weights using [41]:

(1) $$w={\left[2\left(normcdf\left(Z\right)-0.5\right)\right]}^{4}$$

in which *normcdf* is the standard normal cumulative distribution function. Note that the weights are values between 0 and 1.

For each group we obtained a weighted graph in this way. From this graph, we calculated global graph measures (characteristic path length, clustering coefficient, global efficiency and betweenness centrality) as well as local graph measures (node strength, average shortest path length, nodal clustering coefficient, local efficiency and nodal betweenness centrality). These graph measures were calculated using the brain connectivity toolbox (https://sites.google.com/site/bctnet/) (for weighted graphs) except for the (nodal) clustering coefficient and local efficiency, which were calculated using the method described in Wang et al. [41]. The Matlab code used can be accessed through Github (https://github.com/labgas/proj-IBS-somatization). More information about the local graph measures and their interpretation can be found in Box 1.

We also calculated the normalized graph measures defined as the ratio of the graph measure of the network and the mean of the same graph measures obtained in 1000 equivalent random networks. These equivalent random networks are networks with the same number of nodes and the same weight distribution but in which the weights are randomly assigned to the connections.

The hub scores were calculated as the sum of the dummy values for four criteria (each set at 1 or 0 depending on whether or not the criterion is fulfilled, with a maximum hub score of 4) [42,43,44,45]. These criteria are whether the node belongs to the top 20% of nodes:

1. showing the highest strength,
2. showing the lowest path length,
3. showing the lowest local cluster coefficient,
4. showing the highest betweenness centrality.

When a node had a hub score of two or more, it was considered a hub [42].

The modularity structure was determined using the algorithm of Newman [46,47], as implemented in the Brain Connectivity Toolbox, to determine the community structure of the network.

Global and local graph measures were compared between groups using a non-parametric test based on permutation labelling of group membership (5000 realizations). A similar test was used to assess whether the hub score of a node was different between groups. In this analysis, we limited the analysis to nodes which were considered a hub in either of the two IBS groups.

The significantly different covariance were visualized with the BrainNet Viewer (Xia et al., 2013, http://www.nitrc.org/projects/bnv/) [48].

Box 1 **Local graph measures.**

**Node strength** is defined as the sum of weights of all links connected to a specific node [23]. A change in a node with high node strength would strongly affect many other nodes [49].

Paths are sequences of linked nodes. **Path length** in a weighted network is the total sum of individual link length, where link lengths are inversely related to link weights [23]. In anatomical networks, paths represent potential routes of information flow, and shorter paths imply stronger potential integration [23].

The **local clustering coefficient** is a measure of neighborhood connectivity [50], or segregation; the fraction of a node’s neighbors that are neighbors with each other [51]. High clustering is associated with robustness of a network, i.e., resilience against random network damage [52]. Local clustering coefficient and local efficiency are closely related [53].

The efficiency of a network measures how well information propagates over the network [54]. **Local efficiency** is the averaged efficiency of all first-order neighborhoods [55]. The local efficiency of a node is related to the amount of shortest paths that only contains neighbors of the examined node [51]. Local efficiency measures how fault tolerant the system is at a local level, how efficient the communication between neighbors would be if one of the nodes were removed [56].

Centrality regards the relative importance of a node or edge within the overall network architecture, one frequently used metric of centrality is **Betweenness centrality** [57]. Betweenness centrality is defined as the fraction of shortest paths in the network that pass through a given node [23]. Betweenness centrality represent how strongly a given node can influence information flow in the network, an estimate of how a change in a given node would affect the rest of the network [49].

A **hub** is a node with a central position in the overall organization of the network [57]. There are no single measure for defining network hubs, instead it is often preferable to detect hubs by aggregating rankings across different measures, most of which express aspects of node centrality [57].

**Modules** are subgroup of nodes within a network that have stronger connections within the module, and weaker connections to nodes outside of their module [57]. Modules are also called clusters, communities [50], modular structure or community structure [23]. The nodes in a module should have maximally possible within-module connections and minimally possible between-modules connections, and represents a measure of functional segregation [23].

#### 2.6.5. Statistical Analysis

Age, PHQ-14 and TGMV were compared between the three groups using one-way analyses of variance (ANOVA), and the proportion of men/women with a chi-square test on a 2 × 3 contingency table, both with Bonferroni corrected post hoc comparisons of the 3 pairwise between-group differences (Student’s *t*-tests and chi-square test on 2 × 2 contingency tables), in SPSS v24. Significance level for the descriptive statistics was set to *p* < 0.05 (multiple testing corrected for the post-hoc tests).

For the structural covariance and graph analyses we report significance as raw *p*-values < 0.001, while also indicating FDR-corrected *p*-values < 0.05 [58]. As this is, to the best of our knowledge, the first study of gray matter covariance in IBS according to somatization level, we found it suitable to exploratory report the uncorrected results (which should be regarded as hypothesis generating), as well as the more robust findings surviving FDR-correction.

## 3. Results

### 3.1. Population

We included 113 subjects, 77 IBS patients (*n* = 22/55; M/F) and 36 HCs (*n* = 14/22; M/F). Of these, 16 were excluded (11 IBS patients, 5 HCs) due to pathologies found on MRI scan (*n* = 2), another GI disease discovered during the study (*n* = 6), drop-outs (*n* = 3), use of probiotics (*n* = 2), missing values on the PHQ-15 (*n* = 1) and healthy subjects with exclusion criteria (*n* = 2). No subjects were excluded due to poor image quality.

**Figure 1:**
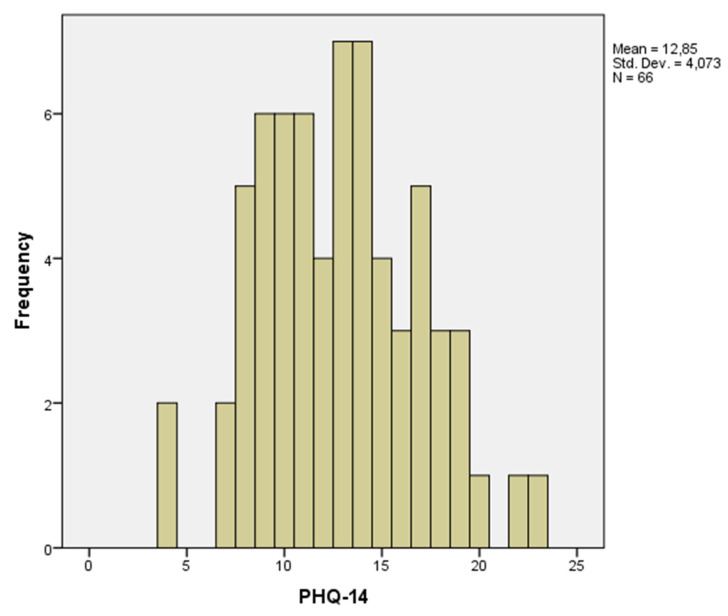
Histogram of the PHQ-14 results in the IBS cohort.

The analyzed population consisted of 66 IBS patients (31 low somatization (*n* = 13/18; M/F) and 35 high somatization (*n* = 5/30; M/F)) and 31 HCs (*n* = 11/20; M/F). The distribution of PHQ-14 in the IBS cohort can be seen in Figure 1. 

There were no differences in age or TGMV between any of the groups. Sex distribution was different between IBS high somatization and IBS low somatization, with significantly more women in the high somatization group. The IBS high somatization group also had a higher proportion of women than the healthy control group, but this difference was not statistically significant. The IBS high somatization group had significantly higher IBS symptom severity measured by IBS-SSS than the low somatization group. The level of psychological distress measured by total HADS score was higher in both IBS groups compared to HCs, and numerically but not significantly higher in the IBS high vs. low somatization group (Table 1).

### 3.2. Structural Covariance Network

#### 3.2.1. IBS High Somatization vs. IBS Low Somatization

**Figure 2:**
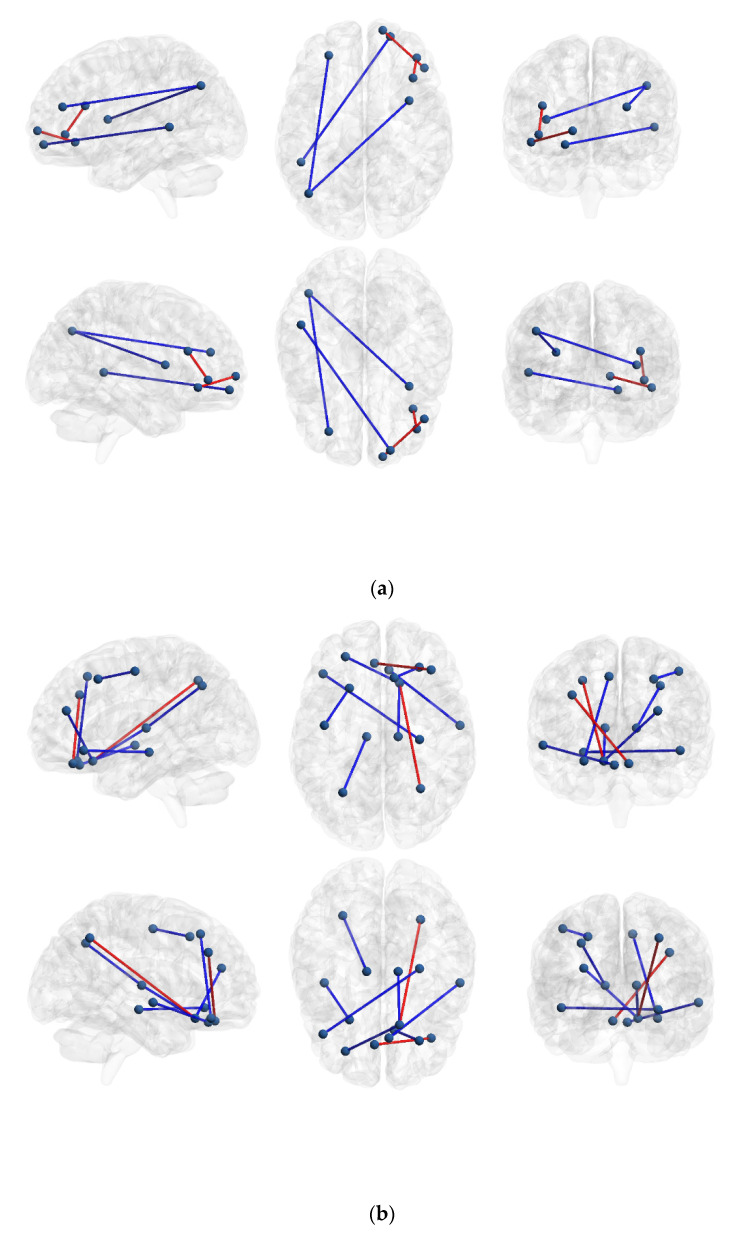

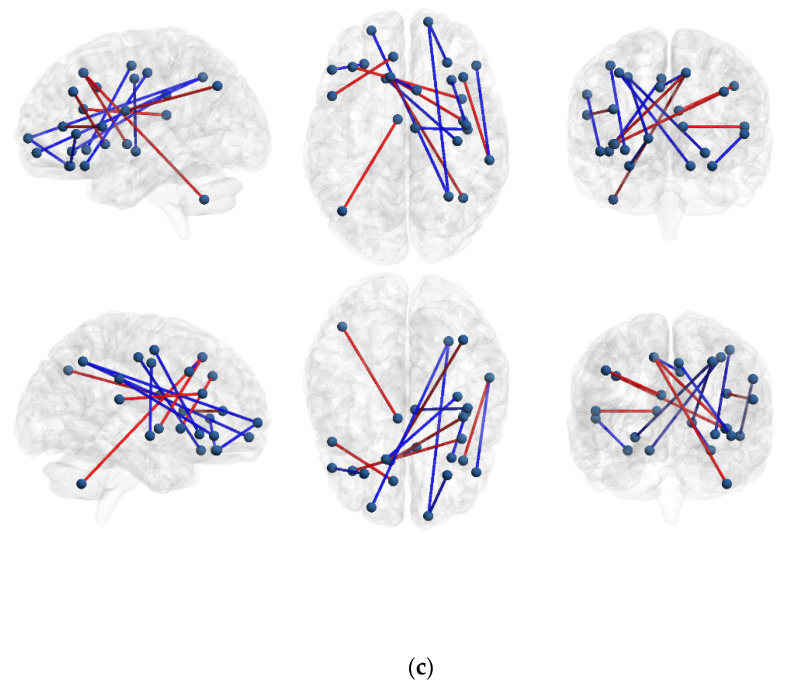
Differences in covariance between groups, significant at *p* < 0.001. (**a**) Differences in covariance between IBS high and low somatization. Covariance greater in IBS high somatization compared to IBS low somatization are shown with red lines, and covariance greater in IBS low somatization compared to IBS high somatization are shown with blue lines. (**b**) Differences in covariance between healthy controls and IBS low somatization. Covariance greater in healthy controls compared to IBS low somatization are shown with red lines, and covariance greater in IBS low somatization compared to healthy controls are shown with blue lines. (**c**) Differences in covariance between healthy controls and IBS high somatization. Covariance greater in healthy controls compared to IBS high somatization are shown with red lines, and covariance greater in IBS high somatization compared to healthy controls are shown with blue lines.

IBS high somatization compared to IBS low somatization had increased structural covariance within parts of the right prefrontal cortex: between the frontal pole and the ventrolateral PFC, and between orbitofrontal cortex and inferior frontal sulcus. IBS low somatization compared to IBS high somatization had increased structural covariance between inferior parietal lobe and middle insula, inferior parietal lobe and middle frontal sulcus of the PFC, and between the frontal pole and superior temporal sulcus (Table 2, Figure 2a). All differences were significant at the uncorrected *p* < 0.001 level, but not at the FDR-corrected *p* < 0.05 significance level.

#### 3.2.2. IBS High and Low Somatization vs. Healthy Controls

Several differences between the two IBS groups and HCs were found at the significance level of uncorrected *p* < 0.001 level, but not at the FDR-corrected *p* < 0.05 significance level. See Supplementary Materials for details (Supplementary Tables S2–S5), and Figure 2b,c. Noteworthy were the increased covariance in IBS high somatization compared to HCs between right frontal pole of the PFC with right orbitofrontal cortex and right inferior parietal cortex respectively, as well as between right inferior parietal and right venterolateral PFC. IBS low somatization had increased covariance between several anterior parts of the prefrontal cortex and thalamus, hippocampus, precentral gyrus (primary somatosensory cortex) and superior temporal gyrus, respectively, compared to HCs.

#### 3.2.3. Graph Analysis

At the global network level, there were no significant differences between the groups.

#### 3.2.4. Local Graph Measures IBS High Somatization vs. IBS Low Somatization

At the local level, IBS high somatization compared to IBS low somatization had increased *clustering coefficients* (reflecting neighborhood connectivity) in the left horizontal ramus of the anterior segment of the lateral sulcus of the prefrontal cortex (clustering coefficient 0.39 vs. 0.22, *p* = 0.0008, not significant after FDR-correction).

#### 3.2.5. Hubs and Hub Scores IBS High Somatization vs. IBS Low Somatization

**Figure 3:**
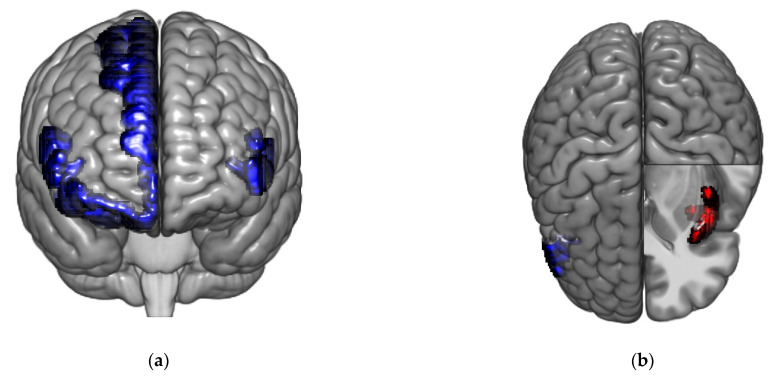
Regions with maximal hub scores in the different groups. Regions belonging to the prefrontal cortex are shown in blue and insular regions are shown in red. (**a**) Regions with maximal hub scores in IBS high somatization. Frontal view of the brain, with the left of the figure being the right of the brain, showing regions with the maximum hub score in IBS high somatization: left and right triangular part of the inferior frontal gyrus, right superior frontal gyrus and right orbital gyri. (**b**) Regions with maximal hub scores in IBS low somatization. Axial view of the brain, with the left of the figure being the right of the brain, showing regions with the maximum hub score in IBS low somatization: right triangular part of the inferior frontal gyrus and left short insular gyri.

In IBS high somatization, several prefrontal cortex subregions were found to be *hubs*, which act as important nodes for the overall network configuration. More specifically, 15 of the 22 hubs were located in the prefrontal cortex, and all regions with the maximum hub score were located in the prefrontal cortex (Figure 3a, Supplementary Table S6).

In IBS low somatization, a mix of regions served as hubs (including parts of anterior and middle insula, prefrontal cortex, putamen and anterior cingulate gyrus) with 11 out of 19 belonging to the prefrontal cortex. The two regions with the maximal hub score were seen in anterior insula and prefrontal cortex (Figure 3b, and Supplementary Table S6).

The most robust group differences were lower hub scores in IBS high somatization compared to IBS low somatization in left cerebellum and left anterior insula (Table 3), both *p* < 0.05 FDR-corrected.

#### 3.2.6. Local Graph Measures IBS High and Low Somatization vs. Healthy Controls

*Local efficiency* in IBS low somatization was higher in left precentral gyrus compared to HCs (high random fault tolerance), *p* < 0.05 FDR-corrected. *Normalized betweenness centrality* was higher in IBS low somatization compared to HCs in the left cerebellum, indicative of increased centrality of left cerebellum in the network in IBS low somatization (Table 4), *p* < 0.05 FDR-corrected. IBS high somatization had increased normalized *path length* of right orbitofrontal cortex of the PFC compared to healthy controls at the uncorrected significance level of *p* < 0.001, not significant after FDR-correction.

#### 3.2.7. Hubs and Hub Scores in Healthy Controls vs. IBS High Somatization and IBS Low Somatization

**Figure 4:**
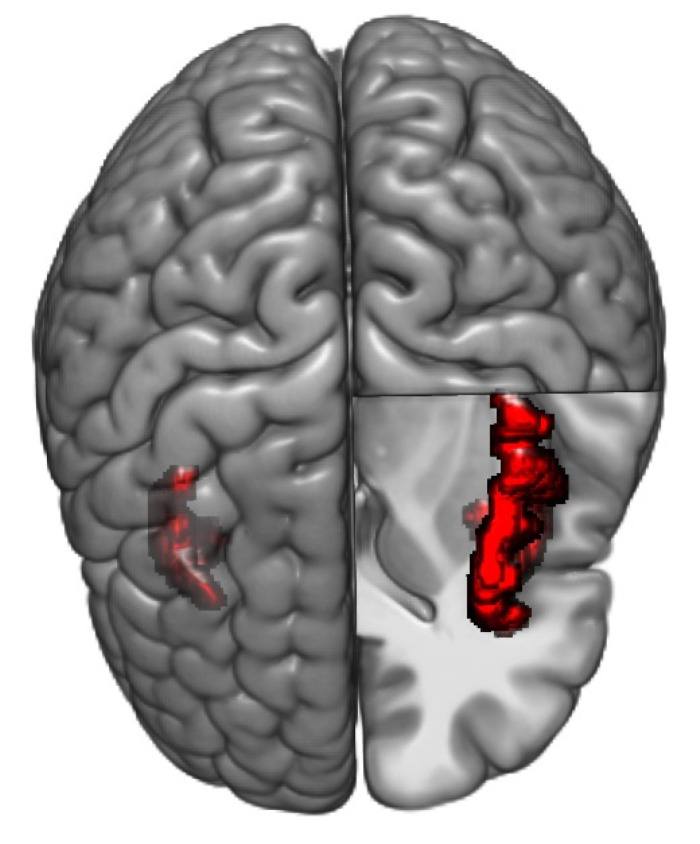
Regions with maximal hub scores in healthy controls: Axial view of the brain, with the left of the figure being the right of the brain, showing regions with the maximum hub score in HCs: left and right short insular gyri and left superior segment of the circular sulcus of the insula.

In HCs, only seven out of 18 hubs were located in the prefrontal cortex, whereas five were located in the insula, including all regions with a maximal hub score (Figure 4 and Supplementary Table S6). In comparison with HCs, IBS high somatization had robustly higher hub scores for parts of prefrontal cortex, and a lower hub score for left anterior insula, both *p* < 0.05 FDR-corrected. IBS low somatization had compared to HCs lower hub scores in parts of insula, and a higher hub score in left cerebellum ( Table 4), all *p* < 0.05 FDR-corrected.

### 3.3. Modular Structure

The modular structure relates to functional segregation and revealed that the 90 brain regions were arranged in eight modules in HCs and IBS low somatization, and nine modules in IBS high somatization (Supplementary Table S7). Of note, both in HCs and IBS low somatization, bilateral amygdala and hippocampus were clustered in the same module. In IBS high somatization, on the contrary, the amygdala clustered with the anterior cingulate cortex, and the hippocampus clustered with the thalamus and parts of bilateral anterior and left mid-posterior insula. There was a distinct cerebellar module in all three groups, but in the IBS low somatization group this was a pure cerebellar module, whilst in IBS high somatization the cerebellum clustered with left transverse frontopolar gyri and sulcus of the prefrontal cortex, and in HCs it clustered with right supramarginal gyrus of the inferior parietal lobe.

## 4. Discussion

This study shows differences in local gray matter structural covariance, defined as correlations between (TGMV corrected) gray matter volumes of regions previously shown to be important to IBS, between IBS with high and low level of somatization, as well as between HCs and both IBS groups.

The most robust findings were: (1) higher centrality and neighbourhood connectivity of prefrontal cortex subregions in IBS high somatization compared to HCs, reflected by higher hub scores and higher clustering coefficient, (2) higher centrality of left cerebellum in IBS low somatization compared to both IBS high somatization and HCs, reflected by higher hub scores and betweenness centrality, and (3) higher centrality of anterior insula in HCs compared to both IBS groups, and in IBS low somatization compared to IBS high somatization, reflected by higher hub scores.

### 4.1. The Novelty of Using Graph Analysis Comparing IBS Subgroups Based on Somatization Level

To the best of our knowledge, there are only two other studies using graph analysis to study structural gray matter covariance in IBS [17,26]. The present study used weighted graphs, with improved methodology [41] compared to the first study which used binary graphs [17]. The other previous study used weighted graphs [26]. The results of weighted graphs retain more information about the network properties as binary graphs only defines a connection as present or absent depending on the chosen threshold(s), whereas weighted graphs bear information about the connection strength between the nodes. However, this is the first study investigating differences in regional gray matter volume covariance between IBS high somatization, IBS low somatization and HCs. Somatization seems to be an important pathophysiological mechanism in IBS. Somatization is, for instance, associated with several measures of increased rectal pain sensitivity in IBS [8]. Including somatization as an important variable of the central nervous system structural covariance network deepens the knowledge on what mechanisms might be involved in the altered brain network organization in IBS.

In line with the study by Labus et al. [17], the overall network organization was intact, reflected by a lack of global differences, whereas there were differences in local graph measures, which quantify the amount of influence (or centrality) of single nodes [51].

The biological attribute of the structural covariance of regional gray matter patterns is not clear, but is thought to be under influence of a complex mixture of developmental, genetic and environmental factors [24]. Evidence supports that structural covariance patterns may arise from genetic influences, mutual trophic reinforcement, experience-related plasticity [59], or could reflect the degree of developmental coordination across the brain [51]. There seem to be substantial but incomplete overlap between structural covariance and white matter connections, as well as functional connectivity [24]. Brain areas that are highly correlated in size are often part of systems that are known to subserve particular behavioural or cognitive functions [24].

If the altered covariance pattern seen in this study is the cause or the consequence of (or possibly a combination of both) experiencing multiple somatic symptoms in IBS remains to be answered. However, we consider the altered covariance pattern a neurobiological substrate involved in the central sensitization process in IBS.

### 4.2. Involvement of the Prefrontal Cortex and Insula

Higher covariance in different prefrontal regions was found in IBS low somatization compared to HCs, and in IBS high somatization compared to IBS low somatization. Further, more hubs in the prefrontal cortex were found in IBS high somatization compared to HCs, as well as a higher hub score in the right orbitofrontal gyrus of the PFC.

This integrative role of hub regions is believed to underlie complex cognitive processes, such as language and abstract thought [60]. A hub region is thought to improve brain function by interacting with many other regions to integrate the associated information [61]. Modelling studies indicate that disruption of hubs is likely to have a particularly deleterious impact on brain network function [60].

The prefrontal cortex constitutes a large portion of the frontal lobe, which is important for performing executive functions [62,63]. The prefrontal cortex is a functionally heterogeneous region, with different subregions having specific characteristics and functions. For example, the orbitofrontal cortex links sensory representations of stimuli to outcomes [64].

Parts of the insula, on the other hand, had higher hub scores in HCs compared to IBS low somatization, and left anterior insula had higher hub scores in both IBS low somatization and HCs compared to IBS high somatization. The role of the insula in visceral sensory processing is well known, with the posterior insular cortex being the primary projection area for visceral afferent information [65]. The anterior insula is implicated in the integration of autonomic and visceral information into emotional, cognitive, and motivational functions [66], as well as for the integration of interoception [66].

In this study, the insula was shown to have a less central role in the covariation network of IBS patients, whereas PFC subregions are more strongly involved in the covariance network in IBS compared to HCs, depending on the level of somatization.

Based on the known functions of the PFC and insula, this might indicate that cognitive-executive aspects are more important than the integration of interoceptive information in IBS, particularly in IBS high somatization. Although highly speculative since it was not tested directly, the results indicates that the evaluation of multiple somatic symptoms is more central to the central sensitization process than increased sensory input seen in IBS with high somatization.

### 4.3. The Importance of the Cerebellum in IBS Low Somatization

The left cerebellum had a more central role in IBS low somatization than both IBS high somatization and HCs. The cerebellum does not only have role in motor functions, but also cognitive functions [67], emotions, social cognition, autonomic functions, perception and pain [68]. Behaviors associated with cerebellar dysfunction have been described as either excessive or reduced responses to the external or internal environment [69]. A role for the cerebellum in disorders of brain-gut-interactions is frequently implicated, but less often discussed in detail. A longitudinal study investigating the prognosis of patients with high frequency migraines using gray matter volumes and structural connectivity found that gray matter volume and structural connectivity of the cerebellum was associated with the headache prognosis two years after the MRI scan [70]. The cerebellum may hypothetically be important for the responsiveness to and persistence of GI symptoms in IBS, but not the more generalized central sensitization process associated with IBS high somatization.

### 4.4. Study Results in Relation to Previous Studies

There are some published studies using graph analysis to study structural covariance in chronic pain disorders. However, the methodology differs in many different ways, rendering direct comparison difficult, and the reasons for disparities between studies are plentiful.

There are some consistent findings between the current study and the previous ones in IBS. In this study, the left triangular part of the inferior frontal gyrus (PFC) was a hub in both IBS high and low somatization but not in HCs, and showed a robust difference in hub scores in IBS high somatization compared to HCs. This is in line with the inferior frontal gyrus being a hub in IBS but not in HC in the previous study by Labus et al. [17]. In the study by Turkiewicz et al. [26], the right medial orbital sulcus (PFC) had higher centrality in IBS than in patients with ulcerative colitis. Increased centrality in IBS compared to this disease control group supports the notion that this alteration might be specific for IBS. An IBS-specific increased centrality of this region is coherent with our study where the right medial orbital sulcus had higher hub scores in IBS high somatization compared to healthy controls and increased covariance with right superior frontal gyrus in IBS low somatization compared to healthy controls. These studies collectively support the central role of PFC subregions in IBS structural covariance networks, compared to disease and non-disease control groups.

Contrary to the study by Labus et al. [17], we did not find the thalamus to be a hub in either IBS groups, but it had increased covariance in the IBS low somatization group compared to HCs with part of the inferior parietal lobe and the medial orbital sulcus of the PFC. They also found the left insular gyrus to be a specific hub in IBS, whereas we found several parts of the insula as having higher hub scores in HCs compared to both IBS groups.

Altered covariance in prefrontal and insular regions in IBS with high and low somatization in our study is in line with the study comparing chronic pelvic pain patients with widespread versus localized symptoms [27].

On the other hand, a study using structural covariance network analysis comparing fibromyalgia patients to HCs found more dense connections in the cerebellum of fibromyalgia patients, while healthy controls exhibited more dense frontal lobe connections [71]. The seemingly opposite results between that study and ours are surprising since fibromyalgia is a condition with widespread pain, hence a prototypical central sensitization syndrome [72]. However, the methodology used in the study by Kim et al. [71] differed substantially from ours. For instance, they used voxel based morphometry, a different anatomical parcellation scheme and used binary graph analysis. Of note is that the node degree differences between fibromyalgia patients and HCs in the prefrontal cortex were at the significance level between *p* < 0.01–<0.04 (uncorrected), whereas the structural covariance connections had significance levels more similar to our (*p* < 0.001).

### 4.5. Clinical Relevance and Implications

IBS is defined by the presence of abdominal pain and disturbed bowel habits, but for a large group of patients with disorders of brain-gut-interactions the non-GI-symptoms negatively influence daily life more than the GI symptoms per se [7]. The majority of the excess in health care costs results from medical care not directly related to lower GI problems [73]. Somatic symptom severity, measured with PHQ-15, shows strong associations with impaired functional status, general health perceptions, increased bodily pain, disability days, symptom-related difficulty and increased consultations with physicians [32]. Further, our group has previously demonstrated that presence of extra-intestinal symptoms may help to define distinct subgroups of IBS patients with relevance for healthcare utilization [74,75]. These facts justify the relevance of investigating somatization in IBS. A firm diagnosis and information is the first line of treatment in IBS [76]. Identifying a “somatization network” provides a neurobiological basis for the classification of IBS (at least when associated with multiple somatic symptoms) as a “central sensitivity syndrome”, as suggested by Yunus [21,72]. This “somatization network” in IBS would include higher centrality of the PFC and lower centrality of the insula. This neurobiological basis would give patients an explanatory model aiding the understanding why they experience symptoms from the gastrointestinal tract, as well as suffer from extra-intestinal symptoms.

### 4.6. Limitations

A limitation of this study is the relatively small sample size. However, it is the first study linking networks of structural covariance measures to somatization in IBS. Our data is from a single site, using volume as gray matter measurement and has a sample size over 30 participants per group, as recommended in a comparability and reliability study on human brain structural covariance analysis [77].

Most of the results were significant at the uncorrected *p* < 0.001 significance level, but only few at the FDR-corrected significant level. We decided to report all results significant at uncorrected *p* < 0.001 since the combination of results at this significance level gives a more complete picture, and these results might serve as hypotheses generating for future studies.

The reported differences in covariance patterns between IBS high and low somatization are unlikely fully explained by psychological distress, as the (small) difference in HADS score between both groups was non-significant. However, the differences seen in both IBS groups compared to HCs could possibly partially be explained by psychological distress.

The relative contribution of general symptom severity versus specific GI symptom severity is hard to disentangle, since somatization level and IBS symptom severity often are associated [78], also in our data (r = 0.43 between IBS-SSS and PHQ-14 scores, *p* < 0.05). The difference between both somatization groups could potentially be influenced by difference in IBS symptom severity, not only the overall somatic symptom burden (i.e., somatization). However, the PHQ-14 scores without the 3 GI questions were significantly different between the IBS low and high somatization group (mean = 5.0 ± 1.9 vs. 10.5 ± 2.6, *p* < 0.0001), showing that the extra-intestinal somatic symptoms differed between the two groups when GI symptoms were not taken into account.

The used cohort includes both sexes but with more females than males as IBS is more common in women. Sex-dependent differences in brain imaging studies of IBS are repeatedly reported [19], and including both sexes in the same cohort is a possible limitation which may interfere with the results. On the other hand, a mixed sex sample is more representative of the general IBS population.

The cross-sectional design of this study limits the possible interpretations regarding causality.

## 5. Conclusions

Somatization level in IBS is related to differences in local gray matter covariance mainly in regions of the prefrontal cortex, insula and cerebellum. This study implicates that prefrontal processes may be more important than insular in the neurobiological sensitization process associated with IBS high somatization.

## Figures and Tables

**Table 1:** Descriptive statistics.

	Healthy Controls	IBS Low Somatization	IBS High Somatization	*p*-Value Omnibus Test
Number of participants	31	31	35	
Age (years)	31.5 ± 9.4	34.1 ± 11.6	31.9 ± 8.1	0.52
Sex (M/F; %F)	11/20, 65%	13/18, 58% §	5/30, 86% §	0.036
PHQ-14	2.8 ± 2.0	9.4 ± 2.0 *#	15.9 ± 2.7 *#	<0.00001
Total gray matter volume (mm^3^)	653,853 ± 64,706	672,120 ± 64,783	638,851 ± 63,168	0.12
IBS-SSS	22.7 ± 26	245 ± 98 *#	320 ± 89 *#	<0.00001
HADS total score	4.61 ± 3.2	13.5 ± 8.0 *	15.9 ± 7.3 *	<0.00001

Results presented as mean ± sd. § = significant difference between IBS low and IBS high somatization at corrected *p* < 0.05. * = significantly different compared to healthy controls at corrected *p* < 0.05. # = significant difference between IBS low and IBS high somatization at corrected *p* < 0.05. F: females, HADS: Hospital Anxiety and Depression scale, IBS-SSS: IBS severity scoring system, M: males, N: number.

**Table 2:** Comparison of structural covariance between IBS patients with high and low somatization.

IBS High Somatization > IBS Low Somatization						
Node Name 1	ROI	Node Name 2	ROI	Z-Score IBS Low	Z-Score IBS High	*p*-Value
R_TrFPoG_S	PFC (frontal pole)	R_InfFGOrp	PFC (vlPFC)	−0.42	0.39	0.0002
R_InfFS	PFC	R_LORs	PFC (OFC)	−0.42	0.44	0.0006
**IBS High Somatization < IBS Low Somatization**						
**Node Name 1**	**ROI**	**Node Name 2**	**ROI**	**Z-Score IBS Low**	**Z-Score IBS High**	***p*-Value**
L_AngG	Inferior parietal	L_MFS	PFC	0.47	−0.35	0.0004
L_AngG	Inferior parietal	R_SupCirInS	mINS	0.41	−0.37	0.0002
L_SupTS	Superior temporal	R_FMarG_S	PFC (frontal pole)	0.002	−0.75	0.0006

Differences based on Fisher r-to-z-transformed bivariate Pearson correlations; significance levels based on permutation labeling with 5000 randomizations. AngG: angular gyrus, FMarG_S: fronto-marginal gyrus (of Wernicke) and sulcus, InfFGOrp: orbital part of the inferior frontal gyrus, InfFS: inferior frontal sulcus, L: left, LORs: lateral orbital sulcus, MFS: middle frontal sulcus, mINS: middle insula, OFC: orbitofrontal cortex, PFC: prefrontal cortex, R: right, SupTS: superior temporal sulcus (parallel sulcus), TrFPoG_S: transverse frontopolar gyri and sulcus, vl: venterolateral.

**Table 3:** Differences in graph measures between IBS high and low somatization.

Graph Measure	Node No.	Node Name	Region	IBS Low	IBS High	*p*-Value
**Clustering coefficient**						
Clustering coefficient;	32	L_ALSHorp	PFC	0.22	0.39	0.0008
**Hub score**						
Hub score	1	L_CeB	Cerebellum	2	1	0.0002 *
Hub score	22	L_ShoInG	aINS	4	0	0.0002 *

All graph measures are at the local/nodal level. Asterisk in the *p*-value column indicates that this group difference is significant at the FDR-corrected *p* < 0.05 level. aINS: Anterior insula, ALSHorp: Horizontal ramus of the anterior segment of the lateral sulcus, CeB: Cerebellum, IBS High: IBS high somatization group, IBS Low: IBS low somatization group, L: Left, R: Right, ShoInG: Short insular gyri.

**Table 4:** Differences in graph measures between IBS high or low somatization on the one hand and healthy controls on the other.

Graph Measure	Node No.	Node Name	Region	HCs	IBS High	IBS Low	*p*-Value
**Path length + normalized**							
Path length (normalized)	88	R_SbOrS	PFC (OFC)	1.05	3.12		0.0006
**Clustering coefficient normalized**							
Clustering coefficient (normalized)	16	L_InfFGOpp	PFC	1.47	2.99		0.0002 *
**Local efficiency**							
Local efficiency	27	L_PRCG	Precentral gyrus (M1)	6 × 10^−^^6^		1.1 × 10^−^^4^	0.0004 *
**Betweenness centrality normalized**							
Betweenness centrality (normalized)	1	L_CeB	Cerebellum	0.000		3.59	0.0002 *
**Hub score**							
Hub score	18	L_InfFGTrip	PFC	0	4		0.0002 *
Hub score	22	L_ShoInG	aINS	4	0		0.0002 *
Hub score	63	R_OrG	PFC (lOFC)	0	4		0.0002 *
Hub score	1	L_CeB	Cerebellum	1		2	0.0002 *
Hub score	38	L_SupCirInS	mINS	4		0	0.0002 *
Hub score	62	R_ShoInG	aINS	4		0	0.0002 *

All graph measures are at the local/nodal level. Asterisk in the *p*-value column indicates that this group difference is significant at the FDR-corrected *p* < 0.05 level. aINS: anterior insula, CeB: cerebellum, IBS High: IBS high somatization, IBS low: IBS low somatization, InfFGOpp: opercular part of the inferior frontal gyrus, InfFGTrip: triangular part of the inferior frontal gyrus, L: left, M1: primary motorcortex, mINS: middle insula, OFC: orbitofrontal cortex, OrG: Orbital gyri, PFC: prefrontal cortex, PRCG: precentral gyrus, R: right, SbOrS: suborbital sulcus (sulcus rostrales, supraorbital sulcus), ShoInG: short insular gyri, SupCirInS: superior segment of the circular sulcus of the insula.

## Data Availability

The data presented in this study are available on request from the corresponding author.

## Supplementary Materials

The following are available online at https://www.mdpi.com/article/10.3390/brainsci11121580/s1, Table S1: Nomenclature, Table S2: Connectivity HC < IBS low somatization, Table S3: Connectivity HC > IBS low somatization, Table S4: Connectivity HC < IBS high somatization, Table S5: Connectivity HC > IBS high somatization, Table S6: Hub scores and Table S7: Modularity structure.
