# Supplementary material for: Altered Structural Covariance of Insula, Cerebellum and Prefrontal Cortex Is Associated with Somatic Symptom Levels in Irritable Bowel Syndrome (IBS)
Source: Brain Sci. 2021 Nov 29;11(12):1580. doi: 10.3390/brainsci11121580 (PMC8699158; doi:10.3390/brainsci11121580)
Supplement: Supplementary file 1 [file brainsci-11-01580-s001.zip › brainsci-1456755-supplementary.pdf]

Node No	Destrieux et al No	Short name subregion	Full name of subregion	Region of interest	Functional subdivision of PFC
1		L_CeB_V	Left Cerebellum Cortex	Cerebellum	
2		L_Tha_V	Left Thalamus Proper	Thalamus	
3		L_Pu_V	Left Putamen	Putamen	
4		L_Hip_V	Left Hippocampus	Hippocampus	
5		L_Amg_V	Left Amygdala	Amygdala	
6		R_CeB_V	Right Cerebellum Cortex	Cerebellum	
7		R_Tha_V	Right Thalamus Proper	Thalamus	
8		R_Pu_V	Right Putamen	Putamen	
9		R_Hip_V	Right Hippocampus	Hippocampus	
10		R_Amg_V	Right Amygdala	Amygdala	
11	1	L_FMarG_S_V	Left Fronto-marginal gyrus (of Wernicke) and sulcus	PFC	Frontal pole
12	5	L_TrFPoG_S_V	Left Transverse frontopolar gyri and sulcus	PFC	Frontal pole
13	6	L_ACgG_S_V	Left Anterior part of the cingulate gyrus and sulcus (ACC)	ACC	
14	7	L_MACgG_S_V	Left Middle-anterior part of the cingulate gyrus and sulcus (aMCC)	aMCC	
15	8	L_MPosCgG_S_V	Left Middle-posterior part of the cingulate gyrus and sulcus (pMCC)	pMCC	
16	12	L_InfFGOpp_V	Left Opercular part of the inferior frontal gyrus	PFC	
17	13	L_InfFGOrp_V	Left Orbital part of the inferior frontal gyrus	PFC	vlPFC
18	14	L_InfFGTrip_V	Left Triangular part of the inferior frontal gyrus	PFC	
19	15	L_MFG_V	Left Middle frontal gyrus (F2)	PFC	dlPFC
20	16	L_SupFG_V	Left Superior frontal gyrus (F1)	PFC	vmPFC/dmPFC/SMA
21	17	L_LoInG_CInS_V	Left Long insular gyrus and central sulcus of the insula	mpINS	
22	18	L_ShoInG_V	Left Short insular gyri	aINS	
23	24	L_OrG_V	Left Orbital gyri	PFC	lOFC
24	25	L_AngG_V	Left Angular gyrus	Inferior parietal lobe	
25	26	L_SuMarG_V	Left Supramarginal gyrus	Inferior parietal lobe	
26	28	L_PosCG_V	Left Postcentral gyrus	Postcentral gyrus	
27	29	L_PRCG_V	Left Precentral gyrus	Precentral gyrus	
28	31	L_RG_V	Left Straight gyrus, Gyrus rectus	PFC	mOFC
29	34	L_SupTGLp_V	Left Lateral aspect of the superior temporal gyrus	Superior temporal gyrus	
30	35	L_PoPl_V	Left Planum polare of the superior temporal gyrus	Superior temporal gyrus	
31	36	L_TPl_V	Left Planum temporale or temporal plane of the superior temporal gyrus	Superior temporal gyrus	

Node No	Destrieux et al No	Short name subregion	Full name of subregion	Region of interest	Functional subdivision of PFC
32	39	L_ALSHorp_V	Left Horizontal ramus of the anterior segment of the lateral sulcus (or fissure)	PFC	
33	40	L_ALSVerp_V	Left Vertical ramus of the anterior segment of the lateral sulcus (or fissure)	PFC	
34	41	L_PosLS_V	Left Posterior ramus (or segment) of the lateral sulcus (or fissure)	Inferior parietal lobe	
35	45	L_CS_V	Left Central sulcus (Rolando's fissure)	Between Pre- and Postcentral gyrus	
36	47	L_ACirIns_V	Left Anterior segment of the circular sulcus of the insula	aINS	
37	48	L_InfCirIns_V	Left Inferior segment of the circular sulcus of the insula	pINS	
38	49	L_SupCirInS_V	Left Superior segment of the circular sulcus of the insula	mINS	
39	52	L_InfFS_V	Left Inferior frontal sulcus	PFC	
40	53	L_MFS_V	Left Middle frontal sulcus	PFC	
41	54	L_SupFS_V	Left Superior frontal sulcus	PFC	
42	55	L_JS_V	Left Sulcus intermedius primus (of Jensen)	Inferior parietal lobe	
43	56	L_IntPS_TrPS_V	Left Intraparietal sulcus (interparietal sulcus) and transverse parietal sulci	Inferior parietal lobe	
44	62	L_LORs_V	Left Lateral orbital sulcus	PFC	OFC
45	63	L_MedOrS_V	Left Medial orbital sulcus (olfactory sulcus)	PFC	OFC
46	64	L_OrS_V	Left Orbital sulci (H-shaped sulci)	PFC	OFC
47	66	L_PerCaS_V	Left Pericallosal sulcus (S of corpus callosum)	ACC/MCC	
48	70	L_SbOrS_V	Left Suborbital sulcus (sulcus rostrales, supraorbital sulcus)	PFC	OFC
49	73	L_SupTS_V	Left Superior temporal sulcus (parallel sulcus)	Superior temporal	
50	74	L_TrTs_V	Left Transverse temporal sulcus	Superior temporal	
51	1	R_FMarG_S_V	Right Fronto-marginal gyrus (of Wernicke) and sulcus	PFC	Frontal pole
52	5	R_TrFPoG_S_V	Right Transverse frontopolar gyri and sulcus	PFC	Frontal pole
53	6	R_ACgG_S_V	Right Anterior part of the cingulate gyrus and sulcus (ACC)	ACC	
54	7	R_MACgG_S_V	Right Middle-anterior part of the cingulate gyrus and sulcus (aMCC)	aMCC	
55	8	R_MPosCgG_S_V	Right Middle-posterior part of the cingulate gyrus and sulcus (pMCC)	pMCC	
56	12	R_InfFGOpp_V	Right Opercular part of the inferior frontal gyrus	PFC	
57	13	R_InfFGOrp_V	Right Orbital part of the inferior frontal gyrus	PFC	vlPFC
58	14	R_InfFGTrip_V	Right Triangular part of the inferior frontal gyrus	PFC	
59	15	R_MFG_V	Right Middle frontal gyrus (F2)	PFC	dlPFC
60	16	R_SupFG_V	Right Superior frontal gyrus (F1)	PFC	vmPFC/dmPFC/SMA
61	17	R_LoInG_CInS_V	Right Long insular gyrus and central sulcus of the insula	mpINS	

Node No	Destrieux et al No	Short name subregion	Full name of subregion	Region of interest	Functional subdivision of PFC
62	18	R_ShoInG_V	Right Short insular gyri	aINS	
63	24	R_OrG_V	Right Orbital gyri	PFC	lOFC
64	25	R_AngG_V	Right Angular gyrus	Inferior parietal lobe	
65	26	R_SuMarG_V	Right Supramarginal gyrus	Inferior parietal lobe	
66	28	R_PosCG_V	Right Postcentral gyrus	Postcentral gyrus	
67	29	R_PRCG_V	Right Precentral gyrus	Precentral gyrus	
68	31	R_RG_V	Right Straight gyrus, Gyrus rectus	PFC	mOFC
69	34	R_SupTGLp_V	Right Lateral aspect of the superior temporal gyrus	Superior temporal gyrus	
70	35	R_PoPl_V	Right Planum polare of the superior temporal gyrus	Superior temporal gyrus	
71	36	R_TPl_V	Right Planum temporale or temporal plane of the superior temporal gyrus	Superior temporal gyrus	
72	39	R_ALSHorp_V	Right Horizontal ramus of the anterior segment of the lateral sulcus	PFC	
73	40	R_ALSVerp_V	Right Vertical ramus of the anterior segment of the lateral sulcus	PFC	
74	41	R_PosLS_V	Right Posterior ramus (or segment) of the lateral sulcus (or fissure)	Inferior parietal lobe	
75	45	R_CS_V	Right Central sulcus (Rolando's fissure)	Between Pre- and Postcentral gyrus	
76	47	R_ACirIns_V	Right Anterior segment of the circular sulcus of the insula	aINS	
77	48	R_InfCirIns_V	Right Inferior segment of the circular sulcus of the insula	pINS	
78	49	R_SupCirInS_V	Right Superior segment of the circular sulcus of the insula	mINS	
79	52	R_InfFS_V	Right Inferior frontal sulcus	PFC	
80	53	R_MFS_V	Right Middle frontal sulcus	PFC	
81	54	R_SupFS_V	Right Superior frontal sulcus	PFC	
82	55	R_JS_V	Right Sulcus intermedius primus (of Jensen)	Inferior parietal lobe	
83	56	R_IntPS_TrPS_V	Right Intraparietal sulcus (interparietal sulcus) and transverse parietal sulci	Inferior parietal lobe	
84	62	R_LORs_V	Right Lateral orbital sulcus	PFC	OFC
85	63	R_MedOrS_V	Right Medial orbital sulcus (olfactory sulcus)	PFC	OFC
86	64	R_OrS_V	Right Orbital sulci (H-shaped sulci)	PFC	OFC
87	66	R_PerCaS_V	Right Pericallosal sulcus (S of corpus callosum)	ACC/MCC	
88	70	R_SbOrS_V	Right Suborbital sulcus (sulcus rostrales, supraorbital sulcus)	PFC	OFC
89	73	R_SupTS_V	Right Superior temporal sulcus (parallel sulcus)	Superior temporal	
90	74	R_TrTs_V	Right Transverse temporal sulcus	Superior temporal	

Supplementary Table S1: Nomenclature

HC < IBS low somatization								
**node no 1**	**node name 1**	**ROI**	**node no 2**	**node name 2**	**ROI**	**Z_HC**	**Z_IBS_low**	**p-value**
2	L_Tha_V	Thalamus	43	L_IntPS_TrPS_V	Inferior parietal lobe	-0,4291	0,4998	0,0002
7	R_Tha_V	Thalamus	85	R_MedOrS_V	PFC (OFC)	-0,571	0,1446	0,0006
9	R_Hip_V	Hippocampus	17	L_InfFGOrp_V	PFC (vlPFC)	-0,664	0,2899	0,0002
27	L_PRCG_V	Precentral gyrus	41	L_SupFS_V	PFC	-0,1265	0,7888	0,0002
40	L_MFS_V	PFC	85	R_MedOrS_V	PFC (OFC)	-0,5613	0,231	0,0004
60	R_SupFG_V	vmPFC/dmPFC/SMA	63	R_OrG_V	PFC (lOFC)	-0,2747	0,4259	0,0008
68	R_RG_V	PFC (mOFC)	69	R_SupTGLp_V	Superior temporal	-0,4714	0,4499	0,0006

Supplementary Table S2: Connectivity HC < IBS low somatization significant at $p < 0.001$. Differences based on Fisher r-to-z-transformed bivariate Pearson correlations; significance levels based on permutation labeling with 5000 randomizations.

HC > IBS low somatization								
**node no 1**	**node name 1**	**ROI**	**node no 2**	**node name 2**	**ROI**	**Z_HC**	**Z_IBS_low**	**p-value**
28	L_RG_V	PFC (mOFC)	59	R_MFG_V	PFC (dlPFC)	0,6716	-0,2629	0,0008
83	R_IntPS_TrPS_V	Inferior parietal lobe	85	R_MedOrS_V	PFC (OFC)	0,5587	-0,4708	0,0002

Supplementary Table S3: Connectivity HC > IBS low somatization significant at $p < 0.001$. Differences based on Fisher r-to-z-transformed bivariate Pearson correlations; significance levels based on permutation labeling with 5000 randomizations.

HC < IBS high somatization								
**node no 1**	**node name 1**	**ROI**	**node no 2**	**node name 2**	**ROI**	**Z_HC**	**Z_IBS_high**	**p-value**
11	L_FMarG_S_V	PFC (frontal pole)	83	R_IntPS_TrPS_V	Inferior parietal	-0,3469	0,4711	0,0006
18	L_InfFGTrip_V	PFC	23	L_OrG_V	PFC (lOFC)	-0,7239	0,0403	0,0004
20	L_SupFG_V	vmPFC/dmPFC/SMA	54	R_MACgG_S_V	aMCC	-0,1519	0,4431	0,0002
45	L_MedOrS_V	PFC (OFC)	75	R_CS_V	Between pre- and postcentral gyrus	-0,4462	0,2417	0,0004
52	R_TrFPoG_S_V	PFC (frontal pole)	63	R_OrG_V	PFC (lOFC)	-0,3324	0,5601	0,0002
52	R_TrFPoG_S_V	PFC (frontal pole)	83	R_IntPS_TrPS_V	Inferior parietal	-0,4113	0,493	0,0008
55	R_MPosCgG_S_V	pMCC	77	R_InfCirIns_V	pINS	-0,2828	0,5291	0,0004
57	R_InfFGOrp_V	PFC (vlPFC)	65	R_SuMarG_V	Inferior parietal	-0,3209	0,3445	0,0008
67	R_PRCG_V	Precentral gyrus	76	R_ACirIns_V	aINS	-0,3492	0,4431	0,0008

Supplementary Table S4: Connectivity HC < IBS high somatization significant at $p < 0.001$. Differences based on Fisher r-to-z-transformed bivariate Pearson correlations; significance levels based on permutation labeling with 5000 randomizations.

HC > IBS high somatization								
**node no 1**	**node name 1**	**ROI**	**node no 2**	**node name 2**	**ROI**	**Z_HC**	**Z_IBS_high**	**p-value**
6	R_CeB_V	Cerebellum	20	L_SupFG_V	vmPFC/dmPFC/SMA	0,076	-0,6709	0,0008
13	L_ACgG_S_V	ACC	16	L_InfFGOpp_V	PFC	0,6127	-0,2131	0,0002
19	L_MFG_V	PFC (dlPFC)	62	R_ShoInG_V	PFC (lOFC)	0,2815	-0,5834	0,0002
20	L_SupFG_V	vmPFC/dmPFC/SMA	61	R_LoInG_CInS_V	mpINS	0,4072	-0,1978	0,0004
24	L_AngG_V	Inferior parietal lobe	47	L_PerCaS_V	ACC/MCC	0,6653	-0,0448	0,0006
71	R_TPl_V	Superior temporal	79	R_InfFS_V	PFC	0,5275	-0,3533	0,0008

Supplementary Table S5: Connectivity HC > IBS high somatization significant at $p < 0.001$. Differences based on Fisher r-to-z-transformed bivariate Pearson correlations; significance levels based on permutation labeling with 5000 randomizations.

IBS low somatization				IBS high somatization				Healthy controls		
Node no	Node name	Hubscore		Node no	Node name	Hubscore		Node no	Node name	Hubscore
22	L_ShoInG_V	4		18	L_InfFGTrip_V	4		22	L_ShoInG_V	4
58	R_InfFGTrip_V	4		58	R_InfFGTrip_V	4		38	L_SupCirInS_V	4
8	R_Pu_V	3		60	R_SupFG_V	4		62	R_ShoInG_V	4
13	L_ACgG_S_V	3		63	R_OrG_V	4		8	R_Pu_V	3
18	L_InfFGTrip_V	3		20	L_SupFG_V	3		52	R_TrFPoG_S_V	3
23	L_OrG_V	3		46	L_OrS_V	3		61	R_LoInG_CInS_V	3
41	L_SupFS_V	3		54	R_MACgG_S_V	3		67	R_PRCG_V	3
56	R_InfFGOpp_V	3		59	R_MFG_V	3		3	L_Pu_V	2
60	R_SupFG_V	3		79	R_InfFS_V	3		12	L_TrFPoG_S_V	2
78	R_SupCirInS_V	3		8	R_Pu_V	2		13	L_ACgG_S_V	2
1	L_CeB_V	2		11	L_FMarG_S_V	2		16	L_InfFGOpp_V	2
16	L_InfFGOpp_V	2		23	L_OrG_V	2		43	L_IntPS_TrPS_V	2
19	L_MFG_V	2		33	L_ALSVerp_V	2		58	R_InfFGTrip_V	2
33	L_ALSVerp_V	2		34	L_PosLS_V	2		71	R_TPl_V	2
35	L_CS_V	2		38	L_SupCirInS_V	2		78	R_SupCirInS_V	2
47	L_PerCaS_V	2		41	L_SupFS_V	2		79	R_InfFS_V	2
59	R_MFG_V	2		52	R_TrFPoG_S_V	2		80	R_MFS_V	2
65	R_SuMarG_V	2		53	R_ACgG_S_V	2		86	R_OrS_V	2
85	R_MedOrS_V	2		55	R_MPosCgG_S_V	2				
				76	R_ACirIns_V	2				
				81	R_SupFS_V	2				
				86	R_OrS_V	2				

Supplementary Table S6: Hub scores

IBS low somatization				IBS high somatization				HC			
**module**	**node no**	**Node name**	**Region**	**module**	**node no**	**Node name**	**Region**	**module**	**node no**	**Node name**	**Region**
module 1	1	L_CeB_V	Cerebellum	module 1	1	L_CeB_V	Cerebellum	module 1	1	L_CeB_V	Cerebellum
	6	R_CeB_V	Cerebellum		6	R_CeB_V	Cerebellum		6	R_CeB_V	Cerebellum
					12	L_TrFPoG_S_V	PFC		65	R_SuMarG_V	Inferior parietal lobe
module 2	2	L_Tha_V	Thalamus								
	7	R_Tha_V	Thalamus	module 2	2	L_Tha_V	Thalamus	module 2	2	L_Tha_V	Thalamus
	11	L_FMarG_S_V	PFC (frontal pole)		4	L_Hip_V	Hippocampus		7	R_Tha_V	Thalamus
	12	L_TrFPoG_S_V	PFC (frontal pole)		7	R_Tha_V	Thalamus		27	L_PRCG_V	Precentral gyrus
	14	L_MACgG_S_V	aMCC		9	R_Hip_V	Hippocampus		67	R_PRCG_V	Precentral gyrus
	15	L_MPosCgG_S_V	pMCC		21	L_LoInG_CInS_V	mpINS				
	19	L_MFG_V	PFC (dlPFC)		22	L_ShoInG_V	aINS	module 3	3	L_Pu_V	Putamen
	26	L_PosCG_V	Postcentral gyrus		42	L_JS_V	Inferior parietal lobe		8	R_Pu_V	Putamen
	27	L_PRCG_V	Precentral gyrus		62	R_ShoInG_V	aINS		12	L_TrFPoG_S_V	PFC (frontal pole)
	35	L_CS_V	Between pre- and postcentral gyrus						14	L_MACgG_S_V	aMCC
	39	L_InfFS_V	PFC	module 3	3	L_Pu_V	Putamen		18	L_InfFGTrip_V	PFC
	41	L_SupFS_V	PFC		8	R_Pu_V	Putamen		19	L_MFG_V	PFC (dlPFC)
	43	L_IntPS_TrPS_V	Inferior parietal lobe						20	L_SupFG_V	vmPFC/dmPFC/SMA
	46	L_OrS_V	PFC (OFC)	module 4	5	L_Amg_V	Amygdala		21	L_LoInG_CInS_V	mpINS
	47	L_PerCaS_V	ACC/MCC		10	R_Amg_V	Amygdala		22	L_ShoInG_V	aINS
	51	R_FMarG_S_V	PFC (frontal pole)		13	L_ACgG_S_V	ACC		24	L_AngG_V	Inferior parietal lobe
	52	R_TrFPoG_S_V	PFC (frontal pole)		53	R_ACgG_S_V	ACC		28	L_RG_V	PFC (mOFC)
	55	R_MPosCgG_S_V	pMCC						32	L_ALSHorp_V	PFC
	59	R_MFG_V	PFC (dlPFC)	module 5	11	L_FMarG_S_V	PFC (frontal pole)		36	L_ACirIns_V	aINS

IBS low somatization				IBS high somatization				HC			
**module**	**node no**	**Node name**	**Region**	**module**	**node no**	**Node name**	**Region**	**module**	**node no**	**Node name**	**Region**
Module 2 cont.	64	R_AngG_V	Inferior parietal lobe	module 5 cont.	14	L_MACgG_S_V	aMCC	module 3 cont.	38	L_SupCirInS_V	mINS
	67	R_PRCG_V	Precentral gyrus		15	L_MPosCgG_S_V	pMCC		41	L_SupFS_V	PFC
	80	R_MFS_V	PFC		17	L_InfFGOrp_V	PFC (vlPFC)		42	L_JS_V	Inferior parietal lobe
	81	R_SupFS_V	PFC		19	L_MFG_V	PFC (dlPFC)		43	L_IntPS_TrPS_V	Inferior parietal lobe
	83	R_IntPS_TrPS_V	Inferior parietal lobe		20	L_SupFG_V	vmPFC/dmPFC/SMA		47	L_PerCaS_V	ACC/MCC
	86	R_OrS_V	PFC (OFC)		23	L_OrG_V	PFC (lOFC)		52	R_TrFPoG_S_V	PFC (frontal pole)
	88	R_SbOrS_V	PFC (OFC)		24	L_AngG_V	Inferior parietal		56	R_InfFGOpp_V	PFC
	89	R_SupTS_V	Superior temporal		25	L_SuMarG_V	Inferior parietal		57	R_InfFGOrp_V	PFC (vlPFC)
	90	R_TrTs_V	Superior temporal		28	L_RG_V	PFC (mOFC)		58	R_InfFGTrip_V	PFC
					32	L_ALSHorp_V	PFC		59	R_MFG_V	PFC (dlPFC)
module 3	3	L_Pu_V	Putamen		36	L_ACirIns_V	aINS		60	R_SupFG_V	vmPFC/dmPFC/SMA
	8	R_Pu_V	Putamen		37	L_InfCirIns_V	pINS		61	R_LoInG_CInS_V	mpINS
					38	L_SupCirInS_V	mINS		62	R_ShoInG_V	aINS
module 4	4	L_Hip_V	Hippocampus		43	L_IntPS_TrPS_V	Inferior parietal		69	R_SupTGLp_V	Superior temporal
	5	L_Amg_V	Amygdala		44	L_LORs_V	PFC (OFC)		73	R_ALSVerp_V	PFC
	9	R_Hip_V	Hippocampus		45	L_MedOrS_V	PFC (OFC)		78	R_SupCirInS_V	mINS
	10	R_Amg_V	Amygdala		46	L_OrS_V	PFC (OFC)		81	R_SupFS_V	PFC
	17	L_InfFGOrp_V	PFC (vlPFC)		47	L_PerCaS_V	ACC/MCC				
	42	L_JS_V	Inferior parietal lobe		50	L_TrTs_V	Superior temporal	module 4	4	L_Hip_V	Hippocampus
					51	R_FMarG_S_V	PFC (frontal pole)		5	L_Amg_V	Amygdala
module 5	13	L_ACgG_S_V	ACC		52	R_TrFPoG_S_V	PFC (frontal pole)		9	R_Hip_V	Hippocampus

IBS low somatization				IBS high somatization				HC			
**module**	**node no**	**Node name**	**Region**	**module**	**node no**	**Node name**	**Region**	**module**	**node no**	**Node name**	**Region**
module 5 cont.	20	L_SupFG_V	vmPFC/dmPFC/ SMA	module 5 cont.	54	R_MACgG_S_V	aMCC	module 4 cont.	10	R_Amg_V	Amygdala
	23	L_OrG_V	PFC (lOFC)		55	R_MPosCgG_S_V	pMCC				
	25	L_SuMarG_V	Inferior parietal lobe		57	R_InfFGOrp_V	PFC (vlPFC)	module 5	11	L_FMarG_S_V	PFC (frontal pole)
	28	L_RG_V	PFC (mOFC)		59	R_MFG_V	PFC (dlPFC)		13	L_ACgG_S_V	ACC
	29	L_SupTGLp_V	Superior temporal		60	R_SupFG_V	vmPFC/dmPFC/ SMA		15	L_MPosCgG_S_V	pMCC
	30	L_PoPl_V	Superior temporal		61	R_LoInG_CInS_V	mpINS		16	L_InfFGOpp_V	PFC
	44	L_LORs_V	PFC (OFC)		63	R_OrG_V	PFC (lOFC)		23	L_OrG_V	PFC (lOFC)
	45	L_MedOrS_V	PFC (OFC)		65	R_SuMarG_V	Inferior parietal		25	L_SuMarG_V	Inferior parietal lobe
	48	L_SbOrS_V	PFC (OFC)		69	R_SupTGLp_V	Superior temporal		29	L_SupTGLp_V	Superior temporal
	50	L_TrTs_V	Superior temporal		76	R_ACirIns_V	aINS		30	L_PoPl_V	Superior temporal
	53	R_ACgG_S_V	ACC		77	R_InfCirIns_V	pINS		31	L_TPl_V	Superior temporal
	54	R_MACgG_S_V	aMCC		78	R_SupCirInS_V	mINS		33	L_ALSVerp_V	PFC
	60	R_SupFG_V	vmPFC/dmPFC/ SMA		80	R_MFS_V	PFC		34	L_PosLS_V	Inferior parietal lobe
	63	R_OrG_V	PFC (lOFC)		83	R_IntPS_TrPS_V	Inferior parietal		37	L_InfCirIns_V	pINS
	66	R_PosCG_V	Postcentral gyrus		85	R_MedOrS_V	PFC (OFC)		39	L_InfFS_V	PFC
	68	R_RG_V	PFC (mOFC)		86	R_OrS_V	PFC (OFC)		40	L_MFS_V	PFC
	69	R_SupTGLp_V	Superior temporal		87	R_PerCaS_V	ACC/MCC		44	L_LORs_V	PFC (OFC)
	71	R_TPl_V	Superior temporal		88	R_SbOrS_V	PFC (OFC)		45	L_MedOrS_V	PFC (OFC)
	74	R_PosLS_V	Inferior parietal lobe		90	R_TrTs_V	Superior temporal		48	L_SbOrS_V	PFC (OFC)
	75	R_CS_V	Between Pre- and postcentral gyrus						49	L_SupTS_V	Superior temporal

IBS low somatization				IBS high somatization				HC			
**module**	**node no**	**Node name**	**Region**	**module**	**node no**	**Node name**	**Region**	**module**	**node no**	**Node name**	**Region**
module 5 cont.	79	R_InfFS_V	PFC	module 6	16	L_InfFGOpp_V	PFC	module 5 cont.	50	L_TrTs_V	Superior temporal
	82	R_JS_V	Inferior parietal lobe		29	L_SupTGLp_V	Superior temporal		51	R_FMarG_S_V	PFC (frontal pole)
	84	R_LORs_V	PFC (OFC)		31	L_TPl_V	Superior temporal		53	R_ACgG_S_V	ACC
	85	R_MedOrS_V	PFC (OFC)		34	L_PosLS_V	Inferior parietal lobe		54	R_MACgG_S_V	aMCC
	87	R_PerCaS_V	ACC/MCC		40	L_MFS_V	PFC		55	R_MPosCgG_S_V	pMCC
					56	R_InfFGOpp_V	PFC		68	R_RG_V	PFC (mOFC)
module 6	16	L_InfFGOpp_V	PFC		71	R_TPl_V	Superior temporal		70	R_PoPl_V	Superior temporal
	18	L_InfFGTrip_V	PFC		74	R_PosLS_V	Inferior parietal lobe		71	R_TPl_V	Superior temporal
	24	L_AngG_V	Inferior parietal lobe		81	R_SupFS_V	PFC		72	R_ALSHorp_V	PFC
	33	L_ALSVerp_V	PFC						74	R_PosLS_V	Inferior parietal lobe
	38	L_SupCirInS_V	mINS	module 7	18	L_InfFGTrip_V	PFC		76	R_ACirIns_V	aINS
	40	L_MFS_V	PFC		30	L_PoPl_V	Superior temporal		77	R_InfCirIns_V	pINS
	49	L_SupTS_V	Superior temporal		33	L_ALSVerp_V	PFC		79	R_InfFS_V	PFC
	56	R_InfFGOpp_V	PFC		39	L_InfFS_V	PFC		80	R_MFS_V	PFC
	61	R_LoInG_CInS_V	mpINS		48	L_SbOrS_V	PFC (OFC)		82	R_JS_V	Inferior parietal lobe
	65	R_SuMarG_V	Inferior parietal lobe		49	L_SupTS_V	Superior temporal		83	R_IntPS_TrPS_V	Inferior parietal lobe
	78	R_SupCirInS_V	mINS		58	R_InfFGTrip_V	PFC		84	R_LORs_V	PFC (OFC)
					70	R_PoPl_V	Superior temporal		85	R_MedOrS_V	PFC (OFC)
module 7	21	L_LoInG_CInS_V	mpINS		72	R_ALSHorp_V	PFC		87	R_PerCaS_V	ACC/MCC
	22	L_ShoInG_V	aINS		73	R_ALSVerp_V	PFC		88	R_SbOrS_V	PFC (OFC)

IBS low somatization				IBS high somatization				HC			
**module**	**node no**	**Node name**	**Region**	**module**	**node no**	**Node name**	**Region**	**module**	**node no**	**Node name**	**Region**
module 7 cont.	32	L_ALSHorp_V	PFC	module 7 cont.	79	R_InfFS_V	PFC	module 5 cont.	90	R_TrTs_V	Superior temporal
	36	L_ACirIns_V	aINS		82	R_JS_V	Inferior parietal				
	37	L_InfCirIns_V	pINS		84	R_LORs_V	PFC (OFC)	module 6	17	L_InfFGOrp_V	PFC (vlPFC)
	57	R_InfFGOrp_V	PFC (vlPFC)								
	58	R_InfFGTrip_V	PFC	module 8	26	L_PosCG_V	Postcentral gyrus	module 7	26	L_PosCG_V	Postcentral gyrus
	62	R_ShoInG_V	aINS		27	L_PRCG_V	Precentral gyrus		35	L_CS_V	Between pre-and postcentral gyrus
	70	R_PoPl_V	Superior temporal		35	L_CS_V	Between pre- and postcentral gyrus		46	L_OrS_V	PFC (OFC)
	72	R_ALSHorp_V	PFC		41	L_SupFS_V	PFC		63	R_OrG_V	PFC (lOFC)
	73	R_ALSVerp_V	PFC		66	R_PosCG_V	Postcentral gyrus		66	R_PosCG_V	Postcentral gyrus
	76	R_ACirIns_V	aINS		67	R_PRCG_V	Precentral gyrus		75	R_CS_V	Between pre-and postcentral gyrus
	77	R_InfCirIns_V	pINS		68	R_RG_V	PFC (mOFC)		86	R_OrS_V	PFC (OFC)
					75	R_CS_V	Between pre- and postcentral gyrus				
module 8	31	L_TPl_V	Superior temporal					module 8	64	R_AngG_V	Inferior parietal lobe
	34	L_PosLS_V	Inferior parietal lobe	module 9	64	R_AngG_V	Inferior parietal lobe		89	R_SupTS_V	Superior temporal
					89	R_SupTS_V	Superior temporal				

Supplementary Table S7: Modularity structure
